# The Interventional Radiology (IR) Gender Gap: A Prospective Online Survey by the Cardiovascular and Interventional Radiological Society of Europe (CIRSE)

**DOI:** 10.1007/s00270-018-1967-3

**Published:** 2018-05-22

**Authors:** Tze Min Wah, Anna Maria Belli

**Affiliations:** 1 Diagnostic and Interventional Radiology Department, Institute of Oncology, St. James’s University Hospital, Leeds Teaching Hospitals NHS Trust, Leeds, LS9 7TF UK; 2 Diagnostic and Interventional Radiology Department, St. George’s University Hospitals NHS Foundation Trust, Blackshaw Road, Tooting, London, SW17 0QT UK

**Keywords:** Interventional radiology, Gender gap survey

## Abstract

**Aim:**

A prospective online survey was conducted by the Cardiovascular Interventional Radiological Society of Europe (CIRSE) to evaluate the gender gap within interventional radiology (IR) and the barriers facing women in IR.

**Materials and Methods:**

A questionnaire (“Appendix”) was devised by the authors and the CIRSE communication and publication team and sent electronically to 750 identifiable female members of CIRSE. Responses were collected from 7 August to 24 August 2017.

**Results:**

The response rate was 19.9% (*n* = 149) with highest responses from UK (18%), Italy (11%), Germany (11%), Spain (7%), Netherlands (5%), France (5%), Sweden (4%), USA (4%). 91% of the respondents were between 31 and 46 years, 83% work full time, 62% spend > 50% of their working time in IR, and 67% practice in a university or tertiary referral institution. 85% were in the minority in their department. 52% had no leadership role in their department, but 67% expressed willingness to consider a leadership position. Their main concerns were work/family life balance, the risks of radiation exposure, the effect of pregnancy on training and practice and the male-dominated work environment.

**Conclusion:**

This survey highlights issues experienced by women in IR. Clear guidance on concerns regarding radiation exposure particularly during pregnancy is needed. Structured and supportive training is required for female IRs who may wish to train or work flexibly. The male-dominated environment is discouraging, and a scheme to promote female IRs would encourage women to take on senior leadership positions and attract more women into the specialty.

## Introduction

Over the last decade, there has been a global drive to increase gender diversity in the workplace in both private and public sectors [1]. There is a wealth of evidence to indicate that organizations with greater gender diversity have better organization, increased financial revenue and are better able to retain talents within the organization [2, 3]. This improvement extends to health care where reports suggest better clinical outcomes by female physicians [4, 5].

The fact that > 50% of medical graduates are female has led to several specialties adjusting their recruitment and training to attract women into what have been perceived as traditionally male-dominated specialties, e.g. surgery [6]. Some have been very successful in increasing the proportion of female trainees, e.g. > 80% of current obstetric and gynaecology trainees are female. However, the proportion of women in interventional radiology remains low at 10% [7].

In an effort to understand the obstacles facing female IRs, CIRSE undertook a survey of its female members.

## Methods

An online questionnaire (“Appendix”) was devised by the authors (T.M.W. and A.M.B.) and sent to 750 female full and junior members of CIRSE that were identifiable as female members in the database. CIRSE is the largest IR society in Europe, and its membership is representative of the specialty in Europe. The data were collected from 7 August to 24 August 2017. The online process was facilitated by the CIRSE communication and publication team (B.R. and R.R.).

## Results

The response rate was 19.9% (*n* = 149) which is the best response for a CIRSE online questionnaire to date.

### Nationality

The percentage responses in descending order from 35 countries worldwide were: UK (18%), Italy (11%), Germany (11%), Spain (7%), Netherlands (5%), France (5%), Sweden (4%), USA (4%) and in the remaining 27 countries (35%) where percentage responses was < 3% in each country.

### Age-Group

The online responses according to age-group were as follows: 30 years or below (6%), 31–45 years (56%), 40–46 years (35%) and > 60 years (3%).

### Work Pattern

83% of respondents worked full time and 11% part time. 8% responded “other” and indicated they were on maternity leave, worked as locums or in research.

The amount of time dedicated to IR was ranked into four categories (Table 1).

**Table 1:** The percentage of IR against the total work time was ranked into four categories

IR: total work time	Percentage of responses (%)	Number of responses
Less than 25%	15	22
25–50%	24	35
51–75%	25	37
More than 75%	37	55

### Healthcare Institution

The majority of respondents worked in a university teaching/tertiary referral hospital (67%; *n* = 101), 21% (*n* = 31) in a general hospital, 9% (*n* = 14) in a private hospital/clinic and 2% (*n* = 3) specified “other”.

The size of the institution was categorized according to their number of hospital beds, and there were five categories (Table 2).

**Table 2:** The size of the institutions according to number of hospital beds

Hospital beds	Percentage of responses (%)	Number of responses
0–49	4.0	6
50–199	4.7	7
200–399	16.1	24
400–799	35.6	53
> 800	39.6	59

The results reflect that most IR practice tends to be centred in large institutions with teaching facilities.

### Gender Ratio

The percentage of female colleagues in the IR department revealed that 69% (*n* = 103) had no or very few female IR colleagues and only 14.7% of the IR departments were female IRs in the majority (Table 3).

**Table 3:** The number of female IR colleagues in department

Composition of female IR in IR department	Percentage of responses (%)	Number of responses
None or only very few	55.7	83
About a quarter	7.4	11
About one-third	8.7	13
About half	6.7	10
About two-thirds	3.4	5
Almost all of them	4.7	7
I am the only one	13.4	20

### Leadership

Respondents were asked whether they had a leadership role in their department. The majority (52%) had no leadership role but 26% said they were team group leaders. As IR is a subspecialty of radiology, this implies that they lead their IR team but are not heads of the Radiology department. However, 12% were departmental heads of department (Table 4).

**Table 4:** The percentage of female IRs with supervisory/leadership roles

Supervisory/leadership roles	Percentage of responses (%)	Number of responses
No	52	78
Yes, Department Head	12	17
Yes, Team/group leader	26	39
Yes, Project leader	10	15

When asked whether their direct superior was male or female, 79%; (*n* = 117) responded that their direct superior was male.

As some leadership roles are not permanent, respondents were asked whether they had ever held a leadership position even if they were not currently in one. 19% (*n* = 29) replied that they had. These roles were wide ranging from CEO/President of a National or International Society to department/university management roles.

### Perceptions

The next part of the survey attempts to quantify respondents’ perceptions of attitudes prevailing within IR.

When faced with the statement “IR is a less attractive career for women than other medical specialties”, 68% of respondents disagreed and 32% agreed. However, when the responses were matched to the different age-groups, female IRs < 45 years were more likely to agree with the statement, particularly those 30 years or less (Table 5).

**Table 5:** “Interventional radiology is a less attractive career for woman than other medical specialties”. Responses according to age-group

	Disagree (%)	Agree (%)
30 years or below	33.3	66.7
31–45 years	59.0	41.0
46–60 years	84.6	15.4
Over 60 years	100.0	0.0

**Fig. 1:**
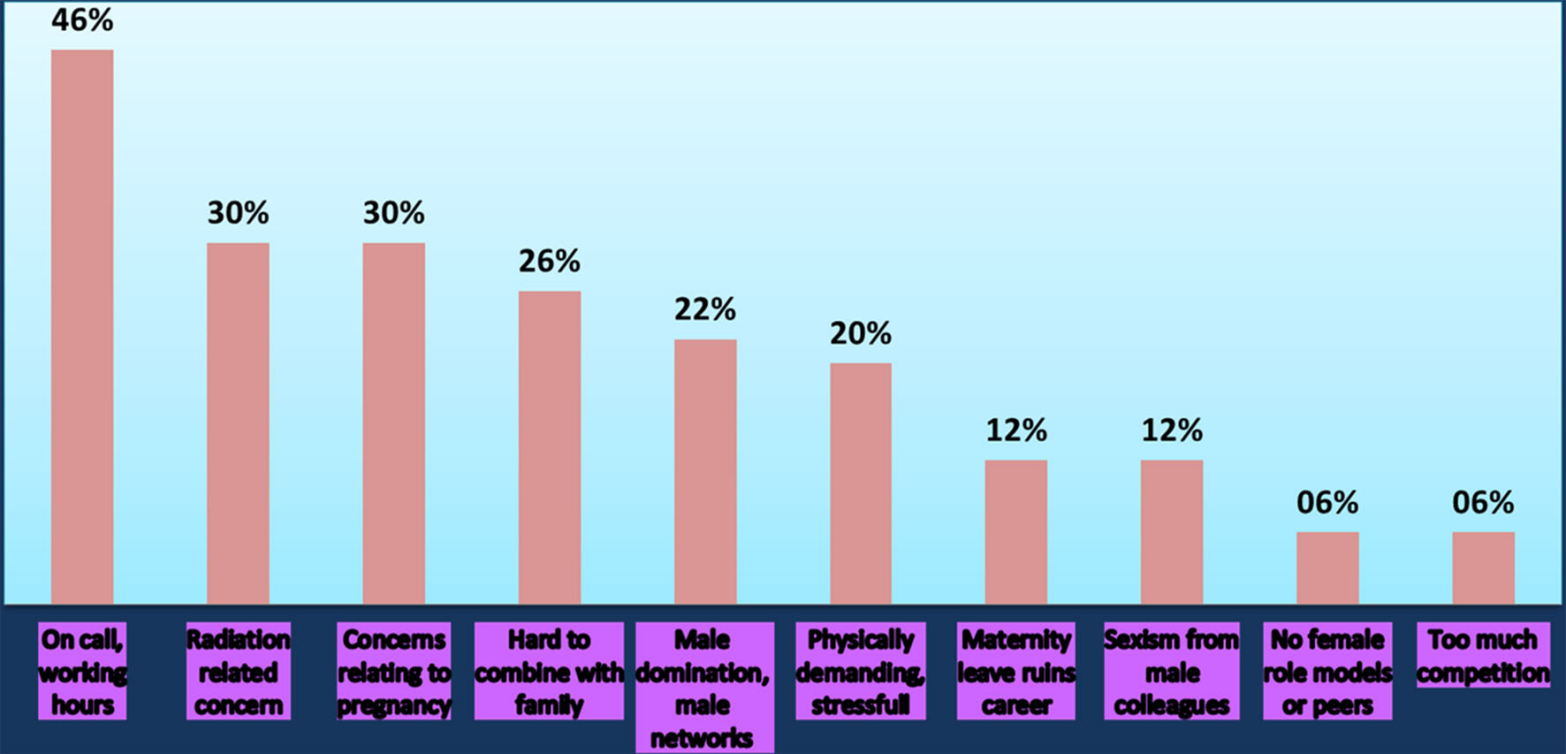
Why do you agree with the following statement? “Interventional radiology is a less attractive career for woman than other medical specialties”

Fifty open responses to this statement were obtained from the respondents (Fig. 1). The top five influencing factors for agreement were: on call working pattern, radiation related concern, pregnancy-related concerns, hard to combine with family and male-dominated network.

In response to the statement “Women are at a disadvantage when pursuing a career in interventional radiology” 63% disagreed whilst 37% agreed. However, when matched to age-group, female IRs < 45 years had a higher percentage likelihood of agreeing with the statement (Table 6).

**Table 6:** “Women are at a disadvantage when pursuing a career in interventional radiology”. Responses matched to age-groups

	Disagree (%)	Agree (%)
30 years or below	55.6	44.4
31–45 years	56.6	43.4
46–60 years	71.2	28.8
Over 60 years	100.0	0.0

**Fig. 2:**
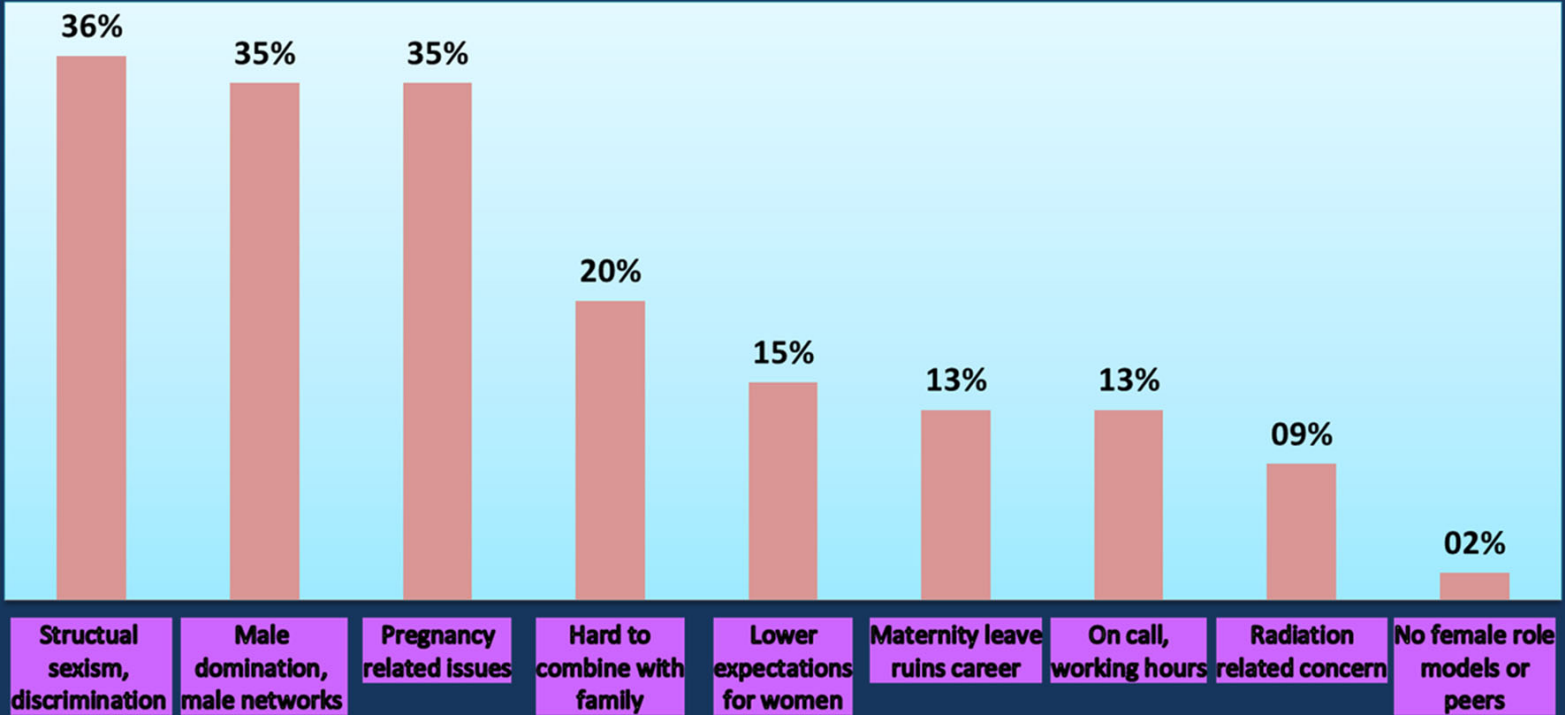
Why do you agree with the following statement? “Women are at a disadvantage when pursuing a career in interventional radiology”

There were 55 open responses in support of this statement. The five main reasons for agreeing with the statement were discrimination, male domination, pregnancy-related issues, hard to combine with family and lower expectations for women (Fig. 2).

In response to the statement “Female IRs and radiologists are treated differently than my male colleagues by my superiors”, 71% of the respondents disagreed and 29% agreed. When analysed according to age-group, only the < 30 years group were more likely to agree with the statement (Table 7). There were 43 open responses, and the four main reasons cited were that female IRs are considered less capable/weak, male-dominated network, unsociable working hours and lower expectation for women.

**Table 7:** “Female IRs and radiologists are treated differently than my male colleagues by my superiors”. Responses matched to age-groups

	Disagree (%)	Agree (%)
30 years or below	44.4	55.6
31–45 years	68.7	31.3
46–60 years	78.8	21.2
over 60 years	80.0	20.0

In response to the statement “As a female IR it is harder to get a promotion”, 72% disagreed, and there was no difference according to age-group (Table 8). A total of 42 open responses were received, and the three main reasons cited by those who agreed with the statement were pregnancy-/maternity-related concern, male-dominated networks, and female IRs are undervalued.

**Table 8:** “As a female IR it is harder to get a promotion”. Responses matched to age-group

	Disagree (%)	Agree (%)
30 years or below	66.7	33.3
31–45 years	68.7	31.3
46–60 years	75.0	25.0
Over 60 years	100.0	0.0

In response to the statement “Female IRs are paid less than their male colleagues”, 88% of respondents disagreed (Table 9).

**Table 9:** Do you agree with the following statement? “Female IRs are paid less than their male colleagues”. Responses matched to age-group

	Disagree (%)	Agree (%)
30 years or below	77.8	22.2
31–45 years	91.6	8.4
46–60 years	84.6	15.4
Over 60 years	80.0	20.0

In response to the statement “It is harder for female IRs to have both a fulfilled career and family life than for male IRs”, 67% agreed with the statement. Female respondents > 45 years were less vehement in their agreement than younger respondents (Table 10).

**Table 10:** “It is harder for female IRs to have both a fulfilled career and family life than for male IRs”. Responses matched to age-groups

	Disagree (%)	Agree (%)
30 years or below	11.1	88.9
31–45 years	27.7	72.3
46–60 years	44.2	55.8
Over 60 years	40.0	60.0

**Fig. 3:**
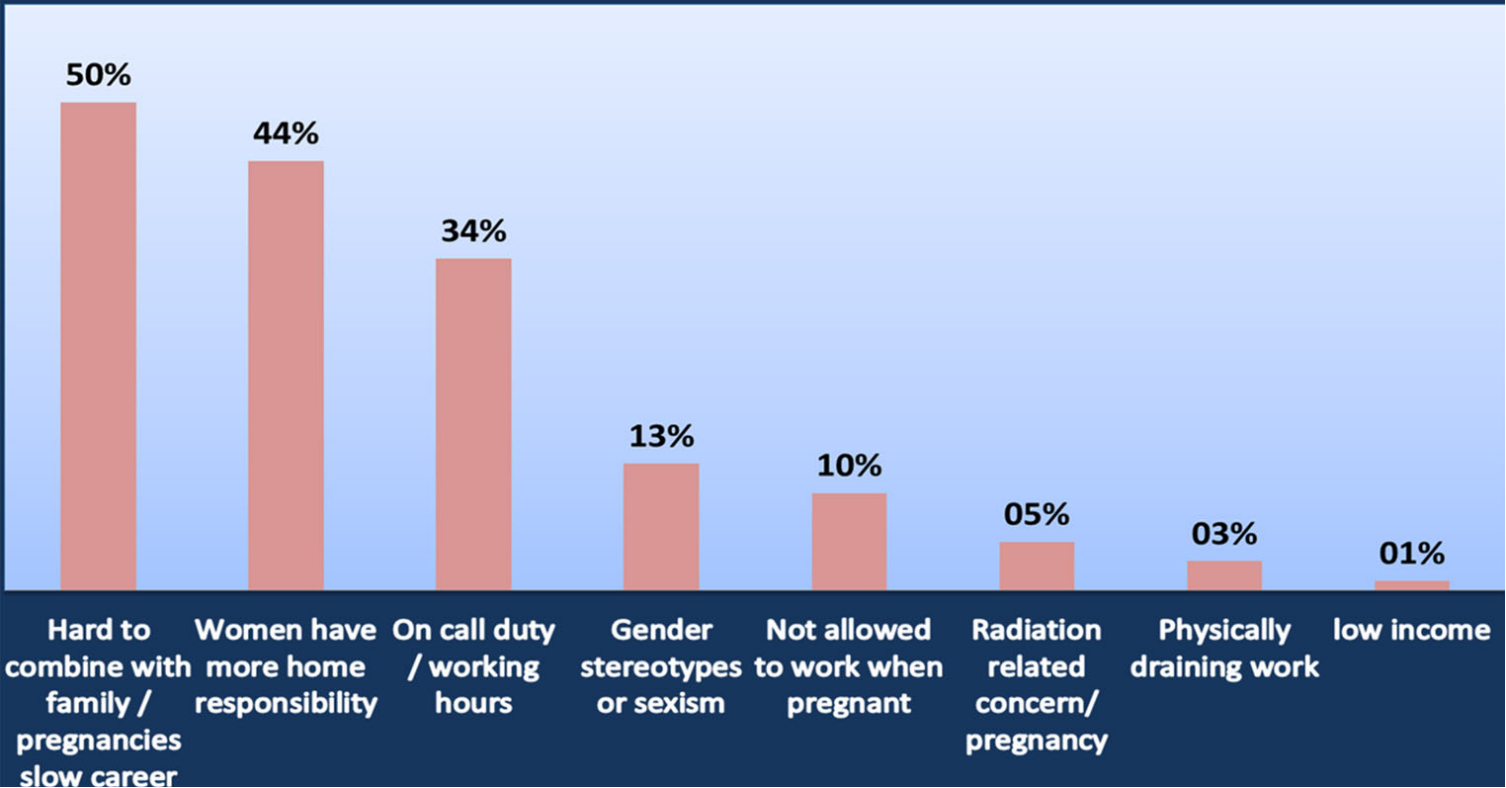
Why do you agree with the following statement? “It is harder for female IRs to have both a fulfilled career and family life than for male IRs” (*n* = 100)

When this statement was matched with the country of origin, the response rate is illustrated in Table 11. There is national variation but only two countries (Denmark and USA) where the majority of respondents disagreed with the statement. One hundred open responses were received and, the three main reasons provided for agreeing with this statement were: difficult to combine with family life as pregnancies slow career progress, women have more domestic responsibilities and on call/long hours working (Fig. 3).

**Table 11:** “It is harder for female IRs to have both a fulfilled career and family life than for male IRs”. Responses matched against the country of origin

	Disagree/strongly disagree (%)	Agree/strongly agree (%)
Austria	25.0	75.0
Denmark	75.0	25.0
France	28.6	71.4
Germany	11.8	88.2
Greece	25.0	75.0
Ireland	50.0	50.0
Italy	29.4	70.6
Netherlands	42.9	57.1
Spain	20.0	80.0
Sweden	16.7	83.3
Turkey	50.0	50.0
United Kingdom (UK)	37.0	63.0
United States of America (USA)	66.7	33.3

**Fig. 4:**
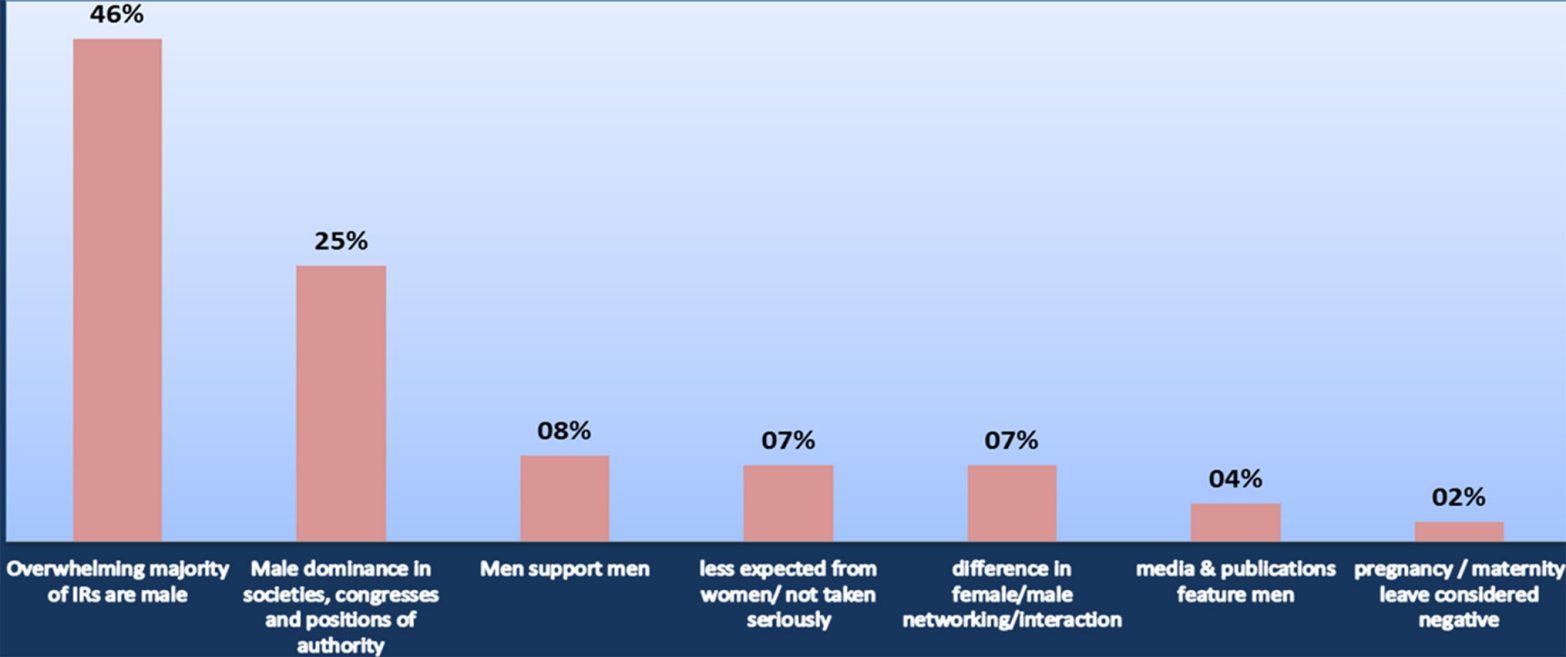
Why do you agree with the following statement? “Interventional radiology is dominated by male networks”

In response to the statement “Interventional radiology is dominated by male networks”, 77% of respondents agreed. Only in the > 60 years age-group did the majority disagree (Table 12). 114 open responses were provided, and the main reason for agreeing with the statement was that the overwhelming majority of IRs are male (Fig. 4).

**Table 12:** Do you agree with the following statement? “Interventional radiology is dominated by male networks”. Responses matched to age-group

	Disagree (%)	Agree (%)
30 years or below	0.0	100.0
31–45 years	20.5	79.5
46–60 years	26.9	73.1
Over 60 years	80.0	20.0

### Mentoring

The majority of respondents (82%; *n* = 107) had a mentor/role model during the early stage of their career. Mentors/role models were 72% male (*n* = 76), 9% female (*n* = 10) and 19% (*n* = 20) had both male and female mentors/role models.

### Role of CIRSE

110 responses were obtained from the open question “What could CIRSE do to make interventional radiology more attractive to female medical students and female radiology trainees?”, and these are illustrated in Table 13.

**Table 13:** What could CIRSE do to make interventional radiology more attractive for female medical students and female radiology trainees? (*n* = 110)

Response category	*n*	%
Support work/training options that allow family life	23	20.9
More female committee members/society leaders	16	14.5
Feature more women in CIRSE publications	15	13.6
Address pregnancy concerns in radiation safety education	15	13.6
Female role model/mentorship programme	15	13.6
More IR exposure at university/increase awareness with students	13	11.8
More female speakers at congresses	13	11.8
General support for female careers	7	6.4
Active promotion of gender equality in IR	7	6.4
Regular women in IR sessions at CIRSE	4	3.6
Female networking events	4	3.6
Grants for female IRs	4	3.6
Educate male colleagues	4	3.6

In response to the final question “Would you, in principle, be interested in holding office for CIRSE or participating in one of its committees or task forces?”, 67% of respondents declared that they would, whilst 6% either have done or currently do so. Only 27% would prefer not to take up a leadership position.

## Discussion

Women are underrepresented in IR. In the UK, although 35% of all radiology consultants are female, only 10% of IR consultants are female [7–9]. 12% of full CIRSE members are female although the numbers of junior members do show a small increase at 18%. More than half of all medical students are women, and it is projected that female doctors working in the National Health Service in the UK will outnumber their male counterparts sometime after 2017 [10]. It is therefore essential for the benefit of patients and the continued delivery of the specialty that IR incorporates an increasing proportion of women into the workforce.

A survey of 143 US female medical students identified the lack of exposure to IR in medical schools, work-related exposure to radiation, length of training, work/life balance and the lack of female role models as barriers to considering a career in IR [11].

Efforts have been made in recent years to raise awareness of IR as a clinical specialty amongst medical students, and a recent US database has shown a larger proportion of female IRs in training (305) when compared to the total number of practicing female IRs (226) [12]. In the USA, direct entry of trainees into vascular surgery rather than via general surgery saw an increase in female vascular surgical trainees from 14 to 38%. With the introduction of integrated IR/DR training in 2016, graduates can enter IR residency directly from medical school, and it is anticipated that this new training programme will have a similar effect [13]. In Europe, entry into IR remains through Diagnostic Radiology.

As confirmed by this survey, the perceived risk from radiation exposure is a particular deterrent for women [14], yet published evidence shows that the occupational radiation exposure to an IR is similar to the natural background radiation dose and continues to decrease with improvements in equipment and good technique [15–17]. So long as the dose remains below the recommended regulatory guidance of < 5 mSv (USA) and < 1 mSv (Europe) throughout pregnancy, there is no increased risk to a foetus when compared to natural background radiation exposure [18]. By way of comparison, flight attendants experience an average annual radiation dose of 1–5 mSv [19] compared with an IR’s average annual dose of 1.6 mSv [15, 17]. Many female IRs continue to work during pregnancy and have foetal radiation doses far below the recommended guidelines. However, health employers and training programme directors who are not always up to date may take a very conservative approach and inadvertently perpetuate the perceived risk by preventing female IRs from continuing their IR training during pregnancy or giving erroneous career advice. This is particularly damaging when imparted by fellow radiologists. This is reflected in the results of the survey as radiation exposure is cited as one of the main concerns for female IRs during their career, especially those < 45 years old.

Work/life balance with on call commitments and the difficulty in combining a career in IR with family life were the other main reasons cited as reasons for IR being perceived as a less attractive career option for women than other specialties. Both are true, but other specialties with onerous on calls and irregular hours manage to attract female trainees. In this survey, most of the respondents (91%) were between 31 and 46 years, i.e. at child bearing age and during the stage of having a young family. Despite this, 83% of them were working full time in a busy IR environment, e.g. university teaching/tertiary referral institutions with > 400 hospitals beds.

When asked whether women were at a disadvantage when pursuing a career in IR, male domination and structural sexism/discrimination were cited as the main reasons for agreeing with this statement. Pregnancy-related issues were also cited and many women reported that they were barred from practicing IR whilst pregnant which along with maternity leave slows their career progress. Younger female IRs (< 30 years) also felt that they were treated differently from their male colleagues by their superiors. Although older female IRs did not agree with this, the results do raise important perceptual differences at a time when aspiring female IRs may be discouraged from pursuing their career.

An interesting result of the survey was the national difference in response to the statement “It is harder for female IRs to have both a fulfilled career and family life than for male IRs”. Although the majority of respondents agreed with this statement for all age-groups, when analysed according to country, the majority of respondents from Denmark and USA disagreed with this statement. This may be a spurious result based on small numbers, but could be due to different social attitudes and training programmes, and this would be worth investigating further.

One of the reasons why women find a career in IR difficult to combine with family responsibilities is the lack of flexible working. In the UK, 40% of all female radiologists work flexibly whilst only 10% of IR consultants work flexibly. In this survey, only 11% of respondents reported that they worked part time. As this survey is directed at female doctors who are already committed to IR, we should question whether the perceived or real lack of flexible working hours prevents female medical graduates from pursuing a career in IR. The fact that respondents aged 30 years or less were the only group where the majority agreed with the statement “IR is a less attractive career for women than other medical specialties” would support this premise. In response to the question “What could CIRSE do to make IR more attractive for female medical students and female radiology trainees?”, 21% of respondents replied “support work/training options that allow family life”.

A repeated theme reported by respondents of this survey was the male domination of the specialty, not surprisingly. Female IRs equaled or outnumbered their male counterparts in 15% of departments with a mere 5% of IR departments being almost exclusively female. With so few women in IR, it is encouraging that there are any female-dominated IR departments. That this is the case suggests that with a significant core of female IR role models, even more women are encouraged to become IRs and are more likely to develop and support creative methods of working. Although male role models were the norm for the women currently practicing IR, the general lack of female role models in leadership positions is problematic. 52% of the respondents have no leadership role, yet the majority (67%) would be interested in holding office for CIRSE or participating in one of its committees or task forces. As there is nothing to prevent these respondents from applying and being elected to such posts, it suggests that female IRs undervalue their skills and need to be approached to apply. An increased number of women holding such positions are likely to encourage more women to pursue an IR career.

## Conclusion

It is heartening that most respondents to this survey disagreed with the statements that IR is a less attractive career option, that it is harder to get promotion as a woman, that women are at a disadvantage or are treated differently from their male counterparts, suggesting that once a career in IR is established women find their career satisfying with equal chances of promotion. However, the hurdle is to attract more women into the speciality.

The results of this survey demonstrate that more needs to be done to educate medical graduates and even practicing radiologists about the facts of radiation exposure during IR procedures so that aspiring female IRs are not deterred.

Although the majority of respondents are in full-time practice, it is likely that they are a self-selected group as it is recognized that female medical graduates are more likely to desire flexible working conditions. IR is perceived, rightly or wrongly, as a full-time commitment, and the lack of availability of flexible training and practice may be putting women off when selecting their career options and steps need to be taken to provide more structured training for those who wish to work flexibly.

The fact that IR involves emergency work and on call is a deterrent to some men as well as women, but other specialties with similar workloads and lifestyles have managed to attract increasing numbers of women and therefore demonstrate that this should not be a major hurdle. However, the fact that entry is via diagnostic radiology may be problematic. Men and women who would be attracted to IR may select other surgical disciplines with a clearer training pathway, whilst those attracted to Diagnostic Radiology may not be those who wish to pursue the lifestyle of IR. The US example of an integrated IR/DR residency will be an interesting model to test whether this theory is correct.

And finally the fact that IR is a male-dominated specialty is a self-fulfilling prophecy. 19% of the respondents have held very senior leadership roles at some point in their career, and 67% expressed an interest in holding a leadership role in principle, yet many fail to do so. Women should be encouraged to apply for such positions and act as role models to inspire the next generation.
